# Case Report: Primary pulmonary lymphoma with CT negative and PET high metabolism combined with hemophagocytic syndrome

**DOI:** 10.3389/fmed.2025.1665417

**Published:** 2025-09-09

**Authors:** Na Dang, Yabo Zhao, Cong Wang, Huifang Ai, Yueqin Chen, Yanhui Wang, Min Du

**Affiliations:** ^1^Department of Medical Imaging, Affiliated Hospital Of Jining Medical University, Jining, China; ^2^Department of Diagnostic Ultrasound, Affiliated Hospital of Jining Medical University, Jining, China; ^3^Department of Pathology, Affiliated Hospital of Jining Medical University, Jining, China

**Keywords:** primary pulmonary lymphoma, PET/CT, hemophagocytic lymphohistiocytosis, case, lung

## Abstract

**Background:**

Primary pulmonary diffuse large B-cell lymphoma (DLBCL) is usually accompanied by imaging visible lesions. Cases with normal CT morphology and high PET metabolism are extremely rare, especially when combined with hemophagocytic syndrome (HLH).

**Case:**

We present the case of a 50-year-old man who presented with fever of unknown origin (FUO) in March 2023. Chest CT showed no abnormalities, while positron emission tomography/computed tomography (PET/CT) showed diffuse hypermetabolism in both lungs (SUVmax 5.1) and slightly increased bone marrow uptake (SUVmax 3.1). Laboratory tests showed a significant increase in ferritin (1132.37 ng / mL) and C-reactive protein (CRP) (115.38 mg/L). Bone marrow smears showed hemophagocytosis. A lung biopsy guided by PET metabolic focus confirmed DLBCL (germinal center type). Complete metabolic remission was achieved after four cycles of R-CEOP chemotherapy.

**Conclusion:**

Primary pulmonary lymphoma (PPL) should be considered in patients with diffuse increased 18F-FDG uptake in both lungs and accompanied by hemophagocytic syndrome when they do not meet the imaging findings of common lung diseases, and the site of increased uptake should be selected for pathological biopsy.

## Introduction

PPL is a rare extranodal lymphoma (1–3), accounting for less than 0.5% of all lymphomas, with DLBCL being particularly uncommon. Patients often present with cough, fever, or dyspnea, and imaging is characterized by pulmonary nodules, consolidation, or interstitial infiltration. When PPL is combined with HLH, the difficulty of diagnosis is significantly increased. As a critical complication of malignant lymphoma, HLH masks the nature of the tumor with an uncontrolled inflammatory storm, resulting in the inability of some patients to determine the cause at the initial diagnosis.

At present, CT remains the core evaluation method for lung lesions, but this case reveals a subversive phenomenon—lung tissue with completely normal CT morphology can harbor metabolically active lymphoma cells. This contradiction stems from the early diffuse interstitial infiltration mode of the tumor—DLBCL cells diffuse along the alveolar septum and have not yet formed a density difference (lower than the CT resolution threshold) but are strongly ingested by ^18^F-FDG due to high expression of the GLUT-1 receptor (SUVmax > 4). More importantly, HLH amplifies the PET signal through a dual mechanism.

## Case report

We present the case of a 50-year-old man who presented with complaints of recurrent fever without an obvious cause for more than 3 months. The fever began 3 months ago, with a maximum temperature of 39.3°C, accompanied by chills and a mild cough, but no rash. Anti-infective treatment during this period was ineffective. A chest CT scan was performed on 6 December 2023, and no significant abnormalities were found. On the same day, laboratory tests were conducted, and the results were as follows: ferritin was significantly increased at 1,132.37 ng/mL (normal range: 21.81–274.66 ng/mL); C-reactive protein was also significantly increased at 115.38 mg/L (normal range: 0–6 mg/L); and both T-spot and Brucella agglutination tests were negative. On 7 December 2023, a bone marrow biopsy was completed, with the following findings: the bone marrow smear showed an increased number of cytoplasmic granules in the granular system, and the phenomenon of hemophagocytosis of tissue cells was occasionally observed, suggesting a possible infection. Irregular lymphocytes were occasionally seen in the biopsy smears. Bone marrow biopsy showed no increase in immature cells or lymphocytes, and megakaryocytes did not exhibit pathological hematopoiesis. Flow cytometry revealed a normal proportion of lymphocytes, with no obvious abnormal lymphocytes found. No abnormal plasma cells were found. Myeloid primitive cells accounted for 0.45%, with no obvious abnormality in phenotype. To determine the cause of the fever, whole-body ^18^F-FDG PET/CT imaging was performed on 8 December 2023.

**Figure 1:**
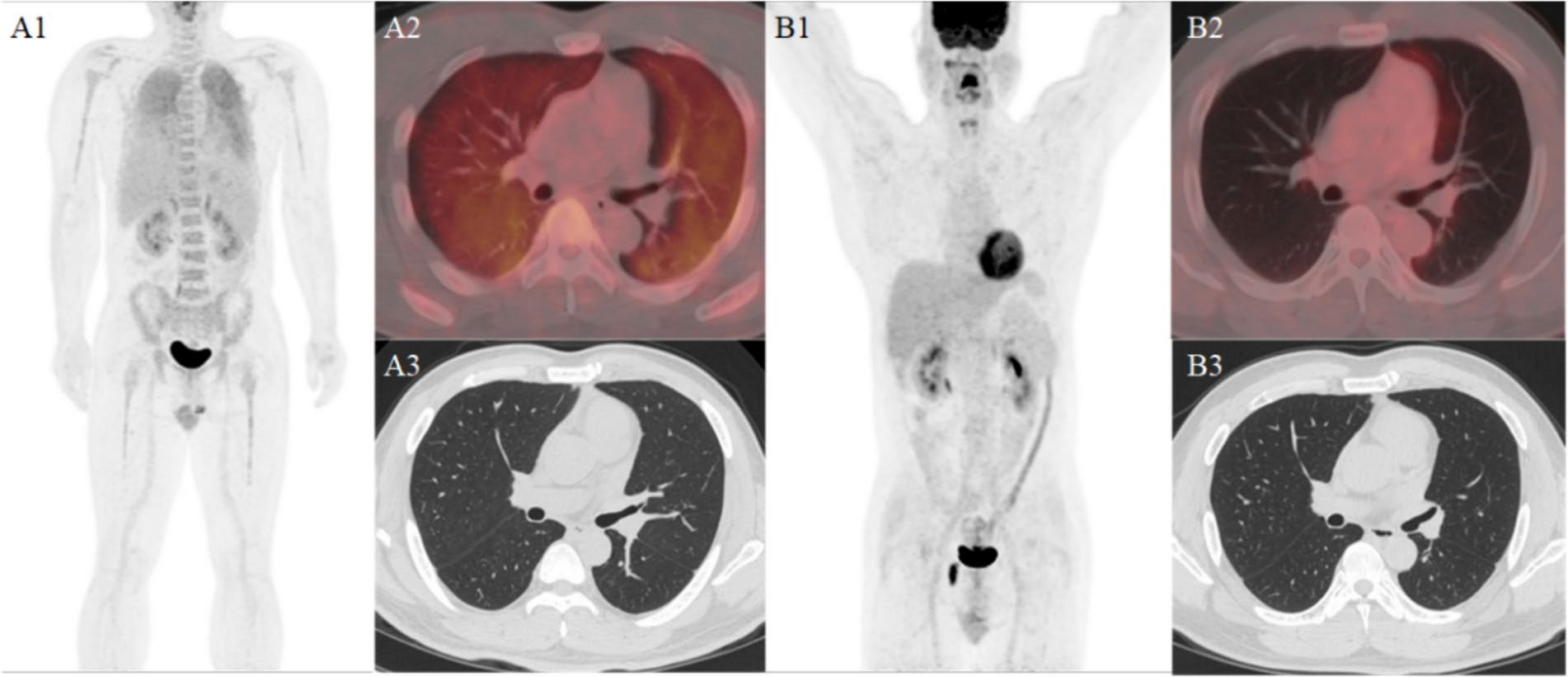
Panel **(A1)** shows diffuse increased metabolism in both lungs and the whole body bones. Panel **(A2)** shows diffuse increased metabolism in both lungs. Panel **(A3)** shows no obvious abnormalities in the high-resolution CT of the same machine. Panel **(B1)** shows no obvious abnormal metabolic increase in the whole body of the patient after treatment. Panel **(B2)** shows and no obvious abnormalities in the PET/CT fusion image. Panel **(B3)** shows and the high-resolution CT of the same machine.

**Figure 2:**
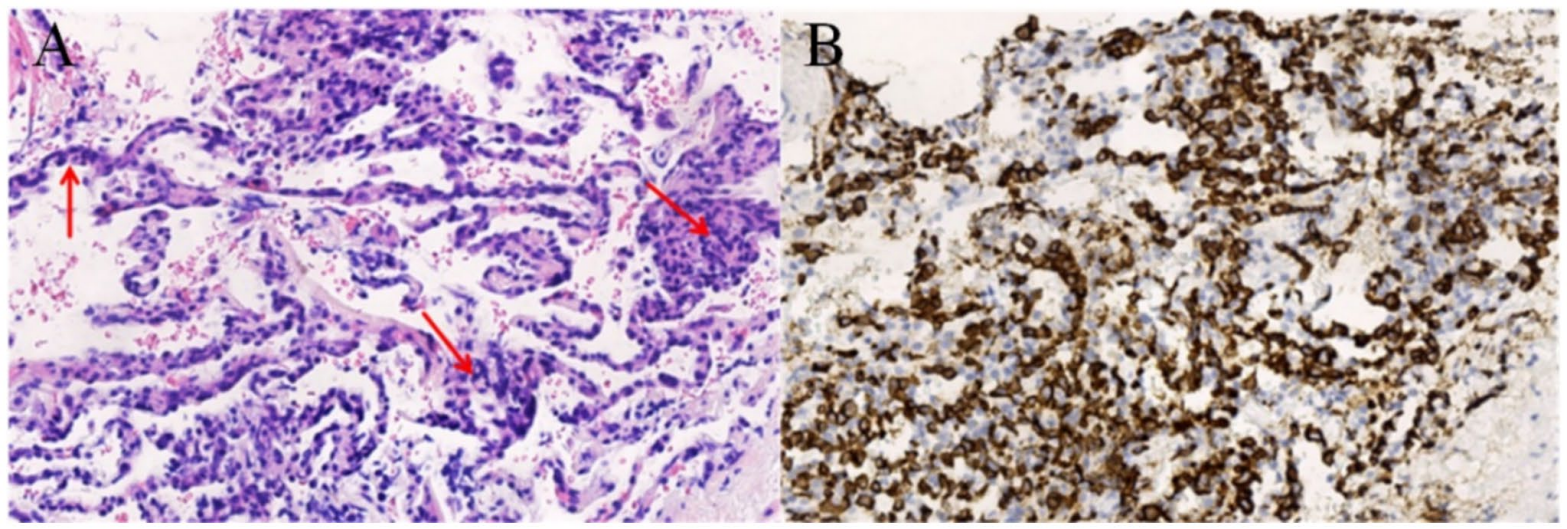
Panel **(A)** HE staining shows that large tumorous B lymphoid cells infiltrated the alveolar wall, and the normal structure of the alveoli was destroyed. Panel **(B)** Immunohistochemistry shows diffuse strong positive expression of B lymphocyte marker CD20 in neoplastic atypical lymphocytes.

The results of ^18^F-FDG PET/CT whole-body imaging were as follows: diffuse increased FDG uptake in both lungs (SUVmax5.1) without obvious nodules and mass shadows on CT and slightly increased diffuse FDG uptake in the proximal bone marrow of the axial bone and limb bones (SUVmax3.1) (see Figure 1). A lung biopsy performed on 19 February 2024 revealed scattered heterotypic large cells, and in combination with immunohistochemistry, it revealed diffuse large B-cell lymphoma (germinal center type). Immunohistochemistry showed TTF-1(+), CK7(−), CK5/6(−), P40(−), CD3(−), CD5(−), CD21(−), CD20(+), CD79a(+), CD10(+), Mum-1(−), Bcl-6(+), Bcl-2(+), and Ki-67(+ 70%) (see Figure 2). Approximately 10 days later, four cycles of chemotherapy with the R-CEOP regimen were administered. After a follow-up whole-body ^18^F-FDG PET/CT scan, the previously noted diffuse FDG metabolism increased shadow. This examination did not show a clear abnormal metabolism increase, suggesting that the activity of the lesion was significantly inhibited or disappeared after treatment. Following four additional cycles of R-CHOP chemotherapy, a third F18-FDG PET/CT scan revealed no significant abnormalities. The clinical team considered this a complete remission, and the patient is currently in good condition (see Figure 1).

## Discussion

PPL is a malignant proliferative disease of lymphocytes involving unilateral or bilateral lungs (parenchyma or bronchi) (4, 5). Primary pulmonary diffuse large B-cell lymphoma is relatively rare in clinical practice. The majority of patients present with cough, dyspnea, fever, and night sweats. In this case, the patients had hemophagocytic syndrome and presented with fever as the first symptom. HLH is a kind of excessive inflammatory response syndrome caused by primary or secondary immune abnormalities, with fever, splenomegaly, and cytopenia as the main symptoms (6). Cytokine storms caused by various reasons, such as infection, autoimmune diseases, and tumors, can all lead to the occurrence of HLH. In adults, HLH is more commonly associated with malignant tumors, while in children, it is often related to infectious lesions. Malignancy-associated hemophagocytic syndrome progresses rapidly, has a poor prognosis, and is challenging to diagnose. Among them, lymphoma-associated hemophagocytic syndrome accounts for the majority, with HLH occurring in approximately 2.8% of lymphoma patients. The two conditions can appear simultaneously or sequentially (7). Early diagnosis and accurate identification of the potential causes of HLH are effective measures to improving prognosis. Therefore, lymphoma screening in HLH patients, especially adults, is the focus of clinical practice. It is believed that the presence of focal FDG uptake in HLH patients during PET/CT imaging is a predictor of malignant tumors, and the SUVmax values of the liver, spleen, and bone in tumor-associated HLH patients are higher in tumor-associated HLH than in non-tumor-associated HLH patients. Studies have also shown that focal bone uptake in patients with HLH suggests the presence of secondary to neoplastic lesions, but focal uptake in the liver and spleen suggests the presence of secondary to infectious lesions (8). The patient in this study showed increased diffuse bone FDG uptake and enlarged spleens, which is consistent with the above reports.

In addition to CT morphological changes, PET/CT also provides information on metabolic activity, molecular characteristics, and receptor distribution, significantly improving the diagnostic accuracy (9, 10). The majority of patients with PPL present with one of the following imaging types: nodular mass type, pneumonia or alveolar type, miliary type, interstitial type, or mixed type, with the nodular mass type being the most common (5). PPL often overlaps with the manifestations of other diseases and can present as inflammatory exudative lesions or solid degeneration lesions similar to pulmonary infections. It can also manifest as interstitial changes similar to those seen in lung cancer. ^18^F-FDG PET/CT imaging can show different degrees of metabolic increase. Peng et al. analyzed that ^18^F-FDG PET/CT can clearly identify the metabolic characteristics and morphology of PPL. There was no obvious abnormality in the CT imaging of the patients in this study, but the diffuse ^18^F-FDG uptake was increased in both lungs, which was rare. The imaging findings of PPL are high metabolism of PET and negative CT, which may be due to the special pathological process of early diffuse interstitial infiltration: at the tumor level, DLBCL cells highly express the GLUT-1 receptor (11) and strongly uptake FDG, resulting in an increase in SUV, but the infiltration of tumor cells along the alveolar septum has not yet formed a density difference, so it is lower than the CT resolution threshold. Second, lymphoma-triggered cytokine storms (IL-6 and IFN-*γ*) activate macrophage hemophagocytosis (12), leading to a sudden increase in ferritin; the latter promotes the exudation of FDG from the blood vessels to the interstitium by increasing pulmonary vascular permeability, further amplifying the PET signal. This process creates a unique diagnostic window period: metabolic abnormalities occur weeks to months earlier than structural changes. This is also one of the factors that the patient had repeated fever for more than 3 months, accompanied by hemophagocytic syndrome, but the cause was not clear for a long time. At the same time, ^18^F-FDG PET / CT imaging can accurately reflect the activity of tumor cells and guide the clinical selection of the optimal puncture biopsy site. In this case, the gold standard of lung biopsy pathology was obtained by targeting the CT-negative PET high metabolic area, leading to a final diagnosis and timely, effective treatment. PET can also exclude secondary pulmonary lymphoma by considering systemic conditions and can be used for staging and treatment efficacy evaluation. In terms of treatment options, the success of the R-CEOP regimen in this case has dual significance: for lymphoma treatment, rituximab combined with chemotherapy directly kills tumor cells. For HLH, etoposide specifically inhibits over-activated macrophages to control the progression of HLH activation in an excessive inflammatory state. This protocol can induce remission treatment while correcting potential immune deficiencies and controlling the primary disease.

## Conclusion

PPL is a rare extranodal lymphoma, and its classic imaging findings are mostly pulmonary nodules or consolidation. However, this case presents an abnormal phenomenon of CT-negative/PET-positive, which reflects the importance of PET examination. In the early stage of some diseases, PET/CT can rely on functional molecular imaging technology to detect abnormalities in time, which is superior to conventional CT or MRI examination.

## Data Availability

The original contributions presented in the study are included in the article/supplementary material, further inquiries can be directed to the corresponding author.
